# The Effect of Semaglutide on Pancreatic β-Cell Function in Adults with Type 2 Diabetes: A Systematic Review and Meta-Analysis

**DOI:** 10.3390/jcm14248734

**Published:** 2025-12-10

**Authors:** Omar Abusedera, Jana Sherif, Malak Smida, Salim Fredericks

**Affiliations:** School of Medicine, Royal College of Surgeons in Ireland-Bahrain, Busaiteen 15503, Bahrain; 23200402@rcsi.com (J.S.); 22200924@rcsi.com (M.S.); sfredericks@rcsi-mub.com (S.F.)

**Keywords:** type 2 diabetes mellitus, semaglutide, pancreatic β-cell function, HOMA-B, HOMA-IR, proinsulin/insulin ratio

## Abstract

**Background/Objectives:** Type 2 diabetes mellitus (T2DM) is characterized by progressive β-cell dysfunction and insulin resistance. Glucagon-like peptide-1 receptor agonists (GLP-1 RAs) may enhance β-cell function. Semaglutide, a long-acting GLP-1 RA, improves glycemic control and weight, but its direct effects on β-cell function remain uncertain. **Methods:** This systematic review and meta-analysis followed PRISMA guidelines and was registered in PROSPERO (CRD420251034071). PubMed, Embase, and Scopus were searched through April 2025 for randomized controlled trials evaluating semaglutide’s effects on β-cell function in adults with T2DM. Primary outcomes included HOMA-B, HOMA-IR, and the proinsulin/insulin ratio; secondary outcomes included insulin secretion rate, insulinogenic index, and C-peptide. Two reviewers independently performed data extraction and risk-of-bias assessment using the Cochrane RoB 1 tool. Random-effects models were used for pooling. Certainty of evidence was evaluated using GRADE. **Results:** Sixteen trials (*n* = 6591) met inclusion criteria, with nine included in the meta-analysis. Semaglutide improved β-cell function (HOMA-B log ratio of means 1.50, 95% confidence interval [CI]: 1.25–1.80) and reduced insulin resistance (HOMA-IR ratio 0.82, 95% CI: 0.73–0.94) compared with placebo or active comparators. The pooled treatment ratio for proinsulin/insulin was 0.70 (95% CI: 0.63–0.79). However, risk of bias was generally high due to open-label designs, and certainty of evidence for all primary outcomes was rated very low. **Conclusions:** Semaglutide appears to improve β-cell function and insulin sensitivity in adults with T2DM, but conclusions remain uncertain given the very low certainty of evidence and substantial heterogeneity. High-quality trials with standardized β-cell outcomes are needed to confirm these findings.

## 1. Introduction

Type 2 diabetes mellitus (T2DM) is a chronic disorder characterized by pancreatic β-cell insulin resistance that occurs primarily in glucose reservoir sites in the body, liver, muscles, and adipose tissues, resulting in glucotoxicity and followed by potential cell impairment and failure [1]. Its prevalence has exponentially increased throughout the past three decades and is estimated to reach 783 million adults by 2045 [2]; hence, preserving β-cell function is key to achieving long-term glycemic control in attempt to significantly alter the disease trajectory on both individual and population levels. Moreover, T2DM pathophysiology is characterized by chronic hyperglycemia, the initial step of non-enzymatic glycation (NEG) that contributes to vascular complications, in addition to oxidative stress and β-cell apoptosis secondary to lipotoxicity associated with hyperglycemia, where increased levels of serum fatty acids are critically reflective of reactive oxygen species (ROS) accumulation. Such mechanisms further reduce insulin synthesis and secretion, mitochondrial function, and cause cell death, leading to the progression from insulin resistance to β-cell destruction [3,4].

Glucagon-like peptide-1 (GLP-1) is an incretin hormone secreted from intestinal L-cells in response to nutrient ingestion. It plays a critical role in glucose homeostasis by stimulating glucose-dependent insulin secretion, inhibiting glucagon release, slowing gastric emptying, and promoting satiety [5,6]; these multifaceted effects not only improve postprandial glycemia but also reduce overall insulin demand, potentially relieving the burden on pancreatic β-cells. Semaglutide is a long-acting GLP-1 receptor agonist (GLP-1 RA), structurally modified to resist degradation by dipeptidyl peptidase-4 (DPP-4), thereby prolonging its half-life and allowing for once-weekly subcutaneous or once-daily oral administration [7,8]. This drug has been approved for T2DM treatment and obesity, with robust clinical evidence supporting its efficacy in reducing HbA1c and promoting sustained weight loss [9]. It may also exert direct and indirect benefits on β-cell function by enhancing glucose-dependent insulin secretion and reducing gluco-toxic stress on β-cells and regulating pancreatic β-cell genes [10].

Semaglutide has been extensively evaluated in large-scale clinical trials for the treatment of T2D, most notably in the SUSTAIN (subcutaneous formulation) and PIONEER (oral formulation) trial programs [11,12,13,14]. Reviews have consistently indicated its efficacy in reducing serum glucose level, body weight, and risk of cardiovascular events [15]. In addition, real-world evidence has similarly demonstrated semaglutide’s effectiveness in routine clinical practice. Several observational cohort studies and registry-based analyses report consistent reductions in HbA1c, body weight, and treatment discontinuation rates among diverse patient populations, further supporting its therapeutic utility outside controlled trial environments [16,17,18,19]. Clinical trials have also reported biomarkers of β-cell function as secondary or exploratory outcomes, such as serum C-peptide levels, HOMA-B (Homeostatic Model Assessment of β-cell function), insulinogenic index, and glucose-stimulated insulin secretion following mixed meal tolerance tests or oral glucose tolerance tests [20].

However, despite the growing interest in β-cell preservation as a therapeutic goal in T2DM, most existing reviews leave a critical gap in understanding how semaglutide may directly modulate β-cell health and functionality through exploring its metabolic effects that may not only enhance short-term glycemic control but also potentially delay the need for treatment intensification, reduce insulin dependence, and alter the natural course of the disease’s progression. This review focuses specifically on semaglutide, assessing its impact on pancreatic β-cell function using validated clinical markers such as C-peptide levels, HOMA-B, insulinogenic index, and stimulated insulin secretion across a broader range of comparators (e.g., placebo, other GLP-1 RAs, insulin, DPP-4 inhibitors) and includes both randomized and non-randomized studies.

## 2. Materials and Methods

This study was conducted according to the Preferred Reporting Items for Systematic Reviews and Meta-Analyses (PRISMA) statement (Table S1) [21]. The protocol was registered in PROSPERO (CRD420251034071).

### 2.1. Data Sources and Search Strategies

A systematic literature search was conducted across three major databases, PubMed, Embase, and Scopus, from inception to 15 April 2025. The search strategy included a combination of controlled vocabulary (e.g., MeSH terms) and free-text keywords to capture both indexed and non-indexed literature relevant to the effect of semaglutide on pancreatic β-cell function in adults with type 2 diabetes. Combined search terms included “semaglutide”, “type 2 diabetes”, and “β-cell function”. No filters were applied, and additional eligible studies were identified by manually screening the reference lists of included articles. Gray literature sources and clinical trial registries were not part of the predefined search strategy, as this review focused on peer-reviewed published studies to ensure methodological consistency and comparable study quality. The full database-specific search strategies are presented in Table S2.

### 2.2. Study Selection

Screening was conducted independently by two of the three reviewers (OA, JS, MS), with any disagreements resolved by the third author. Study selection was conducted through a screening process of two phases using the Covidence platform: title and abstract screening was followed by full-text screening for eligible articles. Artificial intelligence or automated screening tools were not used at any stage of the selection process.

The inclusion criteria for this study were as follows: (1) Studies were randomized controlled trials. (2) Participants were diagnosed with type 2 diabetes, regardless of their body habitus, and were at least 18 years old. (3) The intervention was semaglutide, regardless of the comparator used, as long as the comparator was an approved pharmaceutical agent and not an experimental one. (4) Eligible studies had to report outcomes related to β-cell function. Studies were excluded if they were case reports, editorials, opinion pieces, narrative or systematic reviews, or post hoc analyses. Exceptionally, secondary analyses were included if β-cell function outcomes were pre-specified in the original study. Only full-text, peer-reviewed articles published in English were considered. A list of excluded studies with the primary reason for exclusion is provided in Table S3.

### 2.3. Data Extraction

Data extraction was conducted by two of the three same reviewers who performed the screening, and a third person resolved conflicts. For each included study, we extracted the following data: funding source, study setting, corresponding author details, study start and end dates, eligibility criteria, sample size, number and reasons for participant withdrawal, and baseline characteristics (including age, sex, BMI, and duration of diabetes). Intervention and comparator details, including dosage, duration, and route of administration of semaglutide, were also extracted. Primary outcomes were HOMA-B, HOMA-IR, and proinsulin/insulin ratio. Secondary outcomes included insulin secretion rate, insulinogenic index, β-cell glucose sensitivity, fasting insulin, proinsulin, and C-peptide. Data were extracted at baseline and the latest reported follow-up timepoint. Where baseline and endpoint values were not available, only the reported change from baseline was extracted and used in the synthesis. No artificial intelligence or automated tools were used during this stage of the review.

### 2.4. Risk of Bias Assessment

The risk of bias for each included study was assessed independently by any two reviewers from the three (OA, JS, MS) using the Cochrane Risk of Bias (RoB 1) tool [22]. Overall risk of bias was categorized as “low” if all domains were rated low-risk, “high” if any domain was rated high-risk, and “some concern” in all other cases. Disagreements were resolved by consensus with a third reviewer. No artificial intelligence or automated tools were used during this stage of the review.

### 2.5. Certainty of Evidence

The certainty of evidence for each outcome was evaluated using the GRADE framework [23]. Domains assessed included risk of bias, inconsistency, indirectness, imprecision, and publication bias. Certainty of evidence was categorized as high, moderate, low, or very low according to GRADE guidance. Assessments were conducted independently by any two reviewers from OA, JS, and MS, with disagreements resolved by consensus.

### 2.6. Data Synthesis

For each primary outcome of interest, effect estimates were extracted as a ratio of means (ROM) between intervention and comparator groups at endpoint. When ratios were reported, they were log-transformed for meta-analysis using the generic inverse variance method and then back-transformed for interpretation. *p* < 0.05 (two-tailed) was considered significant. Heterogeneity across studies was evaluated by Cochrane’s Q and I^2^ statistics, considering a P value less than 0.10 or an I^2^ statistic greater than 50% indicative of significant heterogeneity [21]. Studies were grouped by outcome and included in the meta-analysis only if they reported comparable effect measures. In cases where only within-group changes were reported, or where key summary statistics were missing (e.g., standard deviations at endpoint), studies were excluded from pooling and described narratively. All effect estimates were synthesized using a random-effects model, with statistical heterogeneity assessed via I^2^ and Chi^2^ tests. The random-effects analyses used the DerSimonian and Laird method [24]. Subgroup analysis was performed by stratifying doses (0.5 mg and 1.0 mg subcutaneous) together. Sensitivity analyses were conducted by removing outlier studies to test the robustness of pooled estimates. Formal assessment of publication bias using funnel plots or statistical tests was not considered because of the low number of included studies. The risk of publication bias was considered narratively in the context of the available evidence. Primary outcomes were presented as forest plots, and secondary outcomes were only discussed narratively due to lack of studies and severe heterogeneity in results. All statistical analyses were performed using Review Manager V.5.4 statistical software. No artificial intelligence or automated tools were used during this stage of the review.

## 3. Results

### 3.1. Study Characteristics

**Figure 1:**
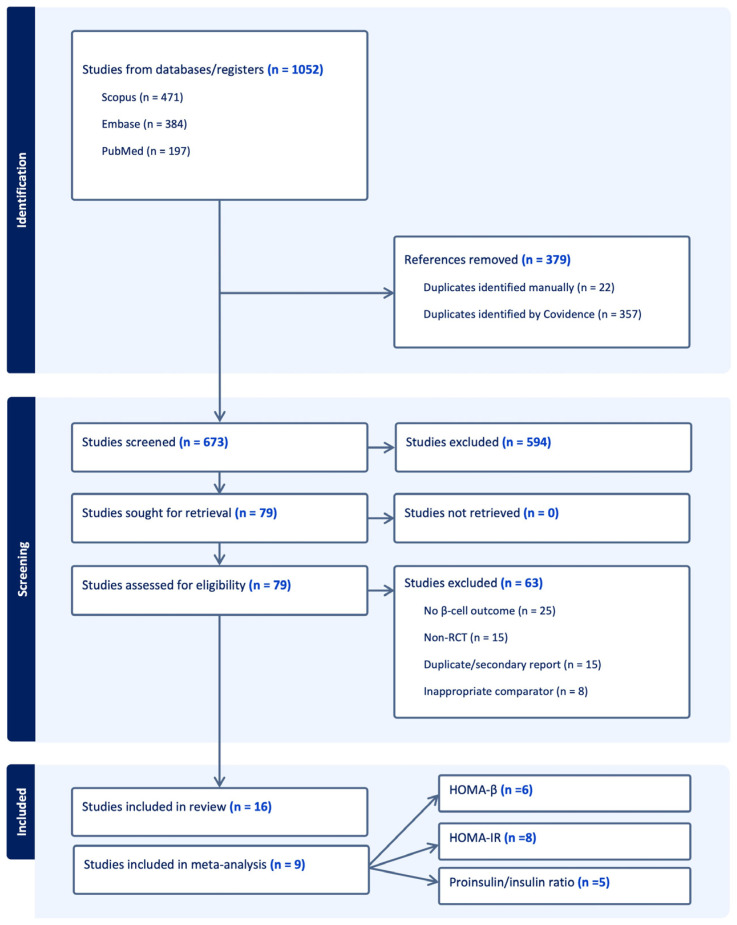
The PRISMA flow diagram for the systematic review detailing the database searches, the number of abstracts screened, and the full texts retrieved.

A total of 1052 studies were imported onto Covidence, and 379 duplicates were found. Seventy-nine articles were then assessed during full text screening. A total of 15 randomized controlled trials and one secondary analysis enrolling 6591 T2D patients were included [25,26,27,28,29,30,31,32,33,34,35,36,37,38,39,40], 9 of which were included for meta-analysis of the three primary outcomes. Figure 1 shows the flowchart for articles selection process. Study and patient characteristics are summarized in Table 1.

Most studies evaluated subcutaneous semaglutide at doses of 0.5 mg or 1.0 mg once weekly (12 studies). Oral semaglutide (10 mg or 14 mg once daily) was used in four studies. In trials where multiple semaglutide doses were tested, data from the FDA-approved 1.0 mg dose were presented. Similar dose arms were evaluated. The SURPASS 2 study compared the hypoglycemic effect of tirzepatide 5 mg, 10 mg, and 15 mg with that of semaglutide 1 mg; therefore, the initial dose of tirzepatide 5 mg was used in this study to compare with semaglutide 1.0 mg. Treatment duration ranged from 12 to 56 weeks, with the majority lasting 24–30 weeks. Comparators included placebo, active agents such as sitagliptin, tirzepatide, exenatide, SGLT2 inhibitors, or insulin aspart, and in some cases both placebo and active controls. One study also evaluated semaglutide in combination with Metformin. Metformin was the most consistent background therapy, either alone or in combination with other oral agents or insulin.

Participants were generally middle-aged adults, with mean ages ranging from 45 to 66 years. The proportion of women varied widely across trials (13–65%), though most studies enrolled balanced cohorts. Baseline BMI ranged from 27 to 34 kg/m^2^, and the duration of diabetes ranged from 3 to over 12 years, with several studies reporting mean durations exceeding 8 years.

Outcomes related to β-cell function and insulin sensitivity included HOMA-B, HOMA-IR, proinsulin/insulin ratio, fasting insulin, proinsulin, insulin secretion rate, insulinogenic index, β-cell glucose sensitivity, and C-peptide. The secondary outcomes were assessed through a range of protocols, including fasting measures, oral glucose tolerance tests (OGTT), mixed meal tolerance tests (MMTT), intravenous glucose tolerance tests (IVGTT), and clamp studies. Therefore, meta-analysis was not considered for these outcomes.

### 3.2. Risk of Bias Assessment

**Figure 2:**
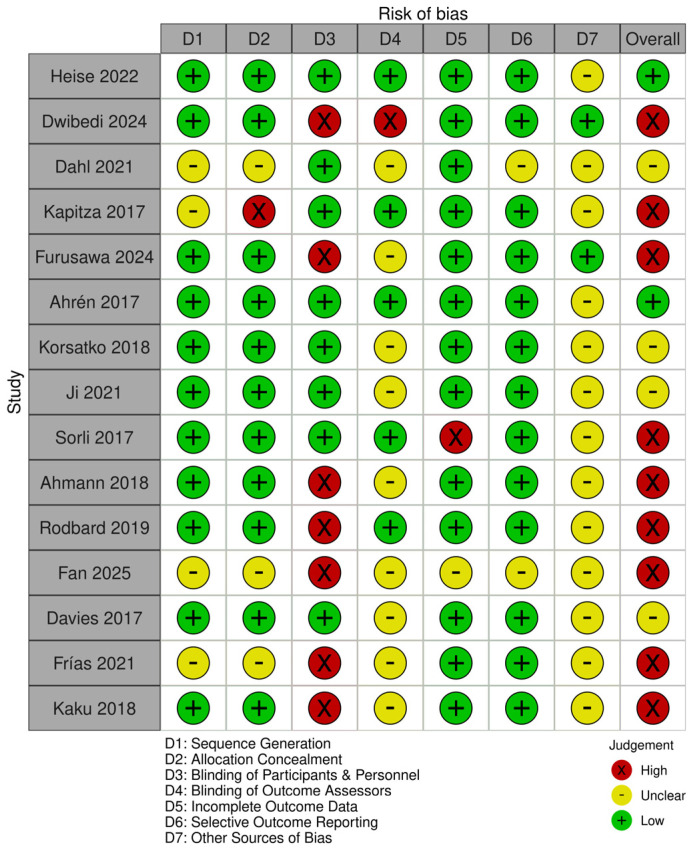
Risk of bias summary showing authors’ judgment for each outcome on each trial, assessed using the Cochrane Risk of Bias (RoB 1) tool [25,26,27,28,29,30,31,32,33,34,35,36,37,38,40].

The risk of bias for the studies was carried out using the Cochrane Risk of Bias (RoB 1) tool [22]. The risk of bias assessment for all trials is shown in Figure 2. Overall, 8/15 studies were double-blinded, and the other 7 were open-label. Only two studies showed low risk of bias, and four showed some concerns. Because less than 10 studies were included in the meta-analysis for each outcome, funnel plots and Egger tests were not used to assess publication bias.

### 3.3. Certainty of Evidence

The GRADE tool was used to assess the certainty of results of the meta-analysis [23]. The tool indicated that the three outcomes had very low certainty of evidence (Table S4). This was due to suspected publication bias, risk of bias from the majority of open-label trials, and inconsistency arising from heterogeneity in results.

### 3.4. Primary Outcomes

#### 3.4.1. Homeostatic Model Assessment of β-Cell Function

**Figure 3:**
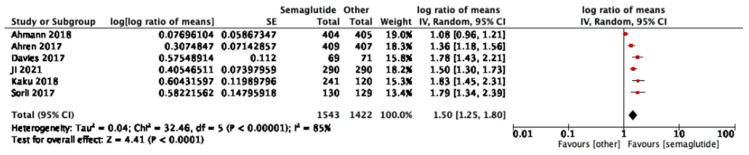
Treatment ratio for HOMA-B between semaglutide and comparators [25,26,29,33,37,40].

Six studies reported HOMA-B outcomes. Semaglutide significantly improved β-cell function compared with comparators (log ratio of means 1.50, 95% CI: 1.25–1.80; I^2^ = 85%) (Figure 3). In subgroup analyses, there was no significant difference between 1.0 mg and 0.5 mg. (*p* = 0.85, I^2^ = 0%) as shown in Figure S1.

#### 3.4.2. Homeostatic Model Assessment for Insulin Resistance

**Figure 4:**
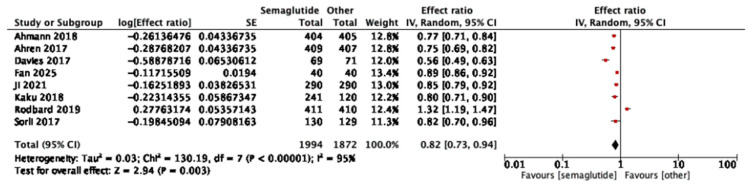
Treatment ratio for HOMA-IR between semaglutide and comparators [25,26,29,30,33,34,37,40].

Eight studies reported HOMA-IR outcomes. Semaglutide reduced insulin resistance compared with comparators (pooled effect ratio 0.82, 95% CI: 0.73–0.94; I^2^ = 95%) (Figure 4). Sensitivity analysis excluding one study [34] reduced heterogeneity to I^2^ = 90% and yielded a pooled effect of 0.77 (95% CI: 0.70–0.85), confirming robustness of the result. In dose-stratified analysis, semaglutide 1.0 mg showed a stronger effect (0.75, 95% CI: 0.68–0.84; six studies, I^2^ = 85%) than 0.5 mg (0.90, 95% CI: 0.87–0.92; five studies, I^2^ = 0%), with a significant subgroup difference (*p* = 0.002, I^2^ = 89.5%) as shown in Figure S2.

#### 3.4.3. Proinsulin/Insulin Ratio

**Figure 5:**
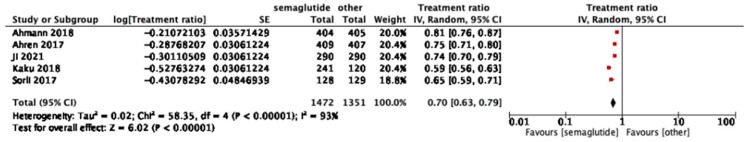
Treatment ratio for proinsulin/insulin between semaglutide and comparators [25,26,33,37,40].

Five studies reported on the proinsulin/insulin ratio. Semaglutide was associated with a significant reduction compared to comparators (pooled treatment ratio 0.70, 95% CI: 0.63–0.79; I^2^ = 93%) (Figure 5). In subgroup analysis by dose, differences were not significant (*p* = 0.78, I^2^ = 0) as shown in Figure S3.

### 3.5. Secondary Outcomes

Three studies evaluated insulin secretion rate (ISR) using IVGTT or MMTT and found consistent improvements with semaglutide compared to placebo or sitagliptin, though tirzepatide produced greater increases in ISR. Two studies assessed β-cell glucose sensitivity and demonstrated significant improvements with semaglutide, while clamp-based analyses suggested larger effects with tirzepatide. One trial reported significant improvements in the insulinogenic index, with stronger effects in obese and GLP-1-naïve patients.

Nine studies reported fasting or stimulated C-peptide, showing generally modest improvements with semaglutide, although findings were inconsistent across comparators. Seven studies assessed fasting insulin, with mixed results depending on disease duration and prior treatment exposure. Four trials consistently demonstrated significant reductions in proinsulin, favoring semaglutide over placebo, sitagliptin, exenatide, and other oral antidiabetic drugs. Finally, two studies investigated disposition index using clamp protocols and found improvements with semaglutide, although tirzepatide showed greater effects.

A detailed summary of all secondary outcomes, including measurement methods, comparators, and direction of effect for each study, is presented in Table S5.

## 4. Discussion

To our knowledge, this is the first systematic review to specifically evaluate semaglutide’s effects on β-cell function in patients with type 2 diabetes. Across 15 randomized trials and one secondary analysis, semaglutide significantly improved β-cell function, with consistent benefits seen across HOMA-B, HOMA-IR, and the proinsulin/insulin ratio. These findings indicate that semaglutide enhances insulin secretory capacity while also reducing markers of insulin resistance, compared with placebo and other glucose-lowering therapies. The results were not significantly different with subgroup analyses by dose. The observed improvements in surrogate markers of β-cell function with semaglutide could be explained by both direct and indirect mechanisms.

The primary goals of diabetes management have always focused on achieving glycemic control, preventing chronic complications, improving quality of life, and prolonging survival. However, preservation and/or restoration of β-cell function has not historically been a central therapeutic target, despite its well-established role in the pathogenesis and progression of T2D [3]. Overall, our results align with literature reviews hypothesizing that GLP-1 agonists can improve beta cell function [41]. Tirzepatide appears to improve beta-cell function more significantly than semaglutide, likely due to its dual receptor function [31,38,39]. Tirzepatide has previously demonstrated its improved glycemic control and weight-loss effects in other meta-analyses [42,43]. Thus, tirzepatide remains the dominant drug overall. In both Dwibedi 2024 and PIONEER 2, where semaglutide increased HOMA-B more than dapagliflozin and empagliflozin, respectively, HOMA-IR reductions favored the SGLT2 inhibitors [28,34]. Both of these studies are reflective of how SGLT2 inhibitors prevent glucose absorption from the kidneys, lowering endogenous insulin secretion [44], whereas semaglutide enhances insulin secretion, which improves β-cell function but can modestly elevate HOMA-IR.

The improvements in HOMA-B, HOMA-IR, and the proinsulin/insulin ratio observed in this review are clinically meaningful, as they suggest that semaglutide not only improves glycemic control but may also help slow the progressive decline of β-cell function that underlies type 2 diabetes. By enhancing insulin secretory capacity and reducing markers of insulin resistance, semaglutide addresses both major components of β-cell dysfunction. These effects go beyond glucose lowering alone, positioning semaglutide as a therapy with potential disease-modifying properties. Importantly, the observed benefits align with semaglutide’s established clinical profile, which includes robust reductions in HbA1c, body weight, and cardiovascular risk. Together, these findings underscore its potential to improve long-term outcomes for patients while also addressing the root pathophysiology of type 2 diabetes.

The findings of this review highlight the need for future large-scale, high-quality randomized trials with standardized β-cell outcome measures to better define the mechanistic and clinical impact of semaglutide on β-cell function. Consistent methodological approaches including harmonized assays, uniform follow-up durations, and predefined metabolic endpoints would improve comparability across studies. From a clinical perspective, semaglutide’s potential to improve β-cell function and reduce insulin resistance suggests a meaningful role in disease-modifying strategies. However, treatment decisions should remain individualized based on each patient’s metabolic profile, comorbidities, disease duration, and risk-benefit considerations.

This systematic review has several strengths: a comprehensive search strategy was employed across multiple databases to ensure no eligible study was overlooked and inclusion criteria were applied rigorously along with dual independent data extraction, avoiding any risks of bias that can interfere with the eligibility of this study, which allows the review to follow a predefined protocol that minimizes selection and analytical bias. This study represents the first systematic effort to focus solemnly on semaglutide in relation to pancreatic β-cell function. The results were generalized because most of the trials were conducted at multiple centers and/or countries. The use of meta-analytic techniques where possible allowed for quantitative synthesis of key endpoints.

However, this review also has important limitations. The included studies demonstrated substantial heterogeneity in the outcomes which is attributed to the differences in the baseline characteristics of included trials, such as race, treatment duration, background medication, control arms, dosage of semaglutide, and the different antidiabetic drugs used as control—a problem faced by a similar review [9]. These factors may modify metabolic responsiveness; for instance, patients on insulin or multi-drug regimens often exhibit more advanced β-cell dysfunction, potentially dampening improvements. Conversely, GLP-1-naïve individuals or those managed with Metformin alone may exhibit greater responsiveness. Such baseline differences complicate cross-trial comparisons and may partially explain the heterogeneity observed in our pooled estimates. Although dose-based subgroup analyses were feasible, the number of available studies per category was insufficient to perform additional subgroup analyses which could have helped explore potential sources of heterogeneity. Some studies lacked sufficient data on outcome measures (e.g., SD) and were therefore excluded from the meta-analysis. Although sensitivity analyses were conducted, the small number of studies within subgroups and substantial heterogeneity may limit the reliability of these findings. Additionally, the gold standard for β-cell function assessment, the clamp-derived disposition index, was only reported in two studies, necessitating reliance on surrogate markers such as HOMA-B, HOMA-IR, and the proinsulin-to-insulin ratio. The risk of bias was overall high or unclear in many trials, particularly due to the lack of blinding, which may reflect challenges in masking subcutaneous treatment. Moreover, all studies except for two were funded by Novo Nordisk and El Lily, which are sponsors for semaglutide and tirzepatide, respectively, which may increase risk of bias. In addition, formal publication bias assessment could not be performed due to the inclusion of fewer than 10 studies per outcome. Nevertheless, trial registries and reference checks were used to reduce this risk; however, these steps were not part of the predefined search strategy as we reviewed them informally during the manuscript revision stage. Finally, GRADE assessment determined the certainty of evidence for all outcomes to be very low, primarily due to study bias, publication bias (automatically downgraded), and inconsistency related to heterogeneity.

## 5. Conclusions

This systematic review and meta-analysis demonstrate that semaglutide enhances β-cell function; however, due to very low certainty of evidence, these findings should be considered hypothesis-generating rather than confirmatory. Beyond glycemic control, semaglutide may represent a therapeutic strategy that preserves β-cell function and delays disease progression, with important implications for long-term management of diabetes. More high-quality trials are needed, however, to confirm our results.

## Figures and Tables

**Table 1:** Baseline characteristics of included studies.

Study ID	NCT	Country	Population	Intervention Group(s)	Control Group(s)	Treatment Duration (Weeks)	Background Therapy	n (Intervention)	n (Control)	Age (Intervention)	Age (Control)	BMI (Intervention)	BMI (Control)	Diabetes Duration (Intervention)	Diabetes Duration (Control)	Outcomes Studied
Ahmann 2018 [26]	NCT01885208	Argentina, Croatia, Finland, France, Germany, Greece, Italy, Netherlands, Serbia, Switzerland, United Kingdom, United States	Adults with T2DM	Semaglutide 1.0 mg s.c.	Exenatide ER 2.0 mg	56	Metformin ± TZD ± SU	404	405	56.4 (NR)	56.7 (NR)	34.0 (NR)	33.6 (NR)	9.0 (NR)	9.4 (NR)	HOMA-B, HOMA-IR, Proinsulin/insulin
Ahrén 2017 [25]	NCT01930188	Argentina, Bulgaria, Czechia, Hong Kong, Hungary, India, Japan, Mexico, Norway, Portugal, Romania, Russia, South Africa, Spain, Sweden, Thailand, Turkey, Ukraine	Adults with T2DM	Semaglutide 0.5 mg s.c.; Semaglutide 1.0 mg s.c.	Sitagliptin 100 mg	56	Metformin ± TZD	409 + 409	407	54.8 ± 10.2; 56.0 ± 9.4	54.6 ± 10.4	32.4 ± 6.2; 32.5 ± 6.6	32.5 ± 5.8	6.4 ± 4.7; 6.7 ± 5.6	6.6 ± 5.1	HOMA-B, HOMA-IR, Proinsulin/insulin
Dahl 2021 [27]	NCT02773381	United Kingdom	Adults with T2DM	Semaglutide 14 mg oral	Placebo	12	Metformin, statins, antihypertensives	15	14	58.2 ± 9.8	58.2 ± 9.8	30.8 ± 2.4	30.8 ± 2.4	3.1 ± 1.8	3.1 ± 1.8	ISR C-peptide β-cell glucose sensitivity
Davies 2017 [29]	NCT01923181	Austria, Bulgaria, Canada, Denmark, Germany, Israel, Italy, Malaysia, Serbia, South Africa, Spain, Sweden, United Kingdom, United States	Adults with T2DM	Semaglutide 10 mg oral; Semaglutide 1 mg s.c.	Placebo	26	Diet/exercise ± Metformin	69 + 69	71	56.5 ± 10.1; 56.8 ± 11.8	58.9 ± 10.3	31.9 ± 4.4; 30.7 ± 4.0	32.6 ± 4.5	5.8 ± 4.8; 5.6 ± 5.0	6.7 ± 5.1	HOMA-B, HOMA-IR
Dwibedi 2024 [28]	NCT04451837	Sweden	Adults with T2DM	Semaglutide	Dapagliflozin	24	Metformin	120	119	65.9 ± 10.0	65.3 ± 9.0	31.2 ± 5.2	31.3 ± 6.0	4.8 ± 3.2	4.3 ± 3.8	HOMA-B, HOMA-IR
Fan 2025 [30]	NR	China	Adults with T2DM	Semaglutide 0.5 mg s.c.	Insulin aspart 30	12	Metformin	40	40	52.4 ± 7.2	53.2 ± 6.8	NR	NR	4.0 ± 1.5	4.1 ± 1.2	HOMA-IR
Frias 2021 [31]	NCT03987919	United States, Argentina, Australia, Brazil, Canada, Israel, Mexico, United Kingdom	Adults with T2DM	Semaglutide 1 mg s.c.	Tirzepatide 5 mg	40	Metformin	469	470	56.9 ± 10.8	56.3 ± 10.0	34.2 ± 7.2	33.8 ± 6.9	8.3 ± 5.8	9.1 ± 7.2	HOMA-IR
Furusawa 2024 [32]	NR	Japan	Adults with T2DM	Semaglutide 14 mg oral	Sitagliptin 100 mg	24	Metformin	75	82	62.2 ± 11.3	63.6 ± 11.9	26.4 ± 4.8	26.1 ± 4.5	NR (>15 years)	NR (>15 years)	HOMA-IR
Heise 2022 [38]	NCT03951753	Germany	Adults with T2DM	Semaglutide 1.0 mg s.c.	Tirzepatide 15 mg; Placebo	28	Metformin	44	45 + 28	63.7 ± 5.9	61.1 ± 7.1; 60.4 ± 7.6	30.8 ± 3.8	31.3 ± 5.0; 32.2 ± 4.0	12.7 ± 6.1	10.2 ± 5.8; 11.0 ± 6.8	Fasting insulin, Total insulin, ISR, Clamp disposition index
Kaku 2018 [40]	NCT02207374	Japan	Adults with T2DM	Semaglutide 0.5 mg s.c.; Semaglutide 1.0 mg s.c.	OAD	56	OAD other than intervention	239 + 240	120	58.0 ± 10.6; 58.7 ± 10.2	59.2 ± 10.1	26.2 ± 4.8; 26.4 ± 4.7	26.7 ± 4.6	8.1 ± 6.0; 9.4 ± 6.5	9.3 ± 7.0	HOMA-B, HOMA-IR, Proinsulin/insulin
Kapitza 2017 [36]	NCT02212067	Germany	Adults with T2DM	Semaglutide 1.0 mg s.c.	Placebo	12	Metformin	37	38	56 (NR)	57 (NR)	29.5 (NR)	29.7 (NR)	8.3 (NR)	8.7 (NR)	C-peptide, Fasting insulin, ISR
Kors atko 2018 [35]	NCT02147431	Austria	Adults with T2DM	Semaglutide 1.0 mg	Placebo	13	Metformin	37	37	54.2 ± 6.4	54.2 ± 6.4	29.4 ± 3.4	29.4 ± 3.4	4.5 ± 3.2	4.5 ± 3.2	C-peptide
Ji 2021 [37]	NCT03061214	Brazil, China, Republic of Korea (South Korea), South Africa, Ukraine	Adults with T2DM	Semaglutide 0.5 mg; Semaglutide 1.0 mg s.c.	Sitagliptin 100 mg	30	Metformin	288 + 290	290	53.0 ± 11.4; 53.0 ± 10.6	53.1 ± 10.4	28.2 ± 5.0; 27.9 ± 5.0	27.3 ± 4.7	6.3 ± 5.4; 6.7 ± 4.9	6.1 ± 5.2	HOMA-B, HOMA-IR, Proinsulin/insulin
Mather 2024 [39]	NCT03951753 (post hoc)	Germany	Adults with T2DM	Semaglutide 1.0 mg s.c.	Tirzepatide 15 mg; Placebo	28	Metformin	44	45 + 28	63.7 ± 5.9	61.1 ± 7.1; 60.4 ± 7.6	30.8 ± 3.8	31.3 ± 5.0; 32.2 ± 4.0	12.7 ± 6.1	10.2 ± 5.8; 11.0 ± 6.8	ISR, β-cell glucose sensitivity
Rodbard 2019 [34]	NCT02863328	Argentina, Brazil, Croatia, Greece, Hungary, Italy, Poland, Russia, Serbia, Spain, Thailand, United States	Adults with T2DM	Semaglutide 14 mg oral	Empagliflozin 25 mg	52	Metformin	411	410	57 ± 10	58 ± 10	32.9 ± 6.3	32.8 ± 5.9	7.2 ± 5.8	7.7 ± 6.3	HOMA-B, HOMA-IR
Sorli 2017 [33]	NCT02054897	Canada, Italy, Japan, Mexico, Russia, South Africa, United Kingdom, United States	Adults with T2DM	Semaglutide 0.5 mg s.c.; Semaglutide 1.0 mg s.c.	Placebo	30	Diet + exercise	128 + 130	129	54.6 ± 11.1; 52.7 ± 11.9	53.9 ± 11.0	32.46 ± 7.62; 33.92 ± 8.43	32.40 ± 6.86	4.8 ± 6.1; 3.6 ± 4.9	4.0 ± 5.5	HOMA-B, HOMA-IR, Proinsulin/insulin

Legend: BMI—body mass index (kg/m^2^); HOMA-B—Homeostatic Model Assessment of β-cell function; HOMA-IR—Homeostatic Model Assessment of insulin resistance; OAD—oral antidiabetic drug; s.c.—subcutaneous; ER—extended release; NR—not reported.

## Data Availability

The original contributions presented in this study are included in the article/Supplementary Materials. Further inquiries can be directed to the corresponding author.

## Supplementary Materials

The following supporting information can be downloaded at: https://www.mdpi.com/article/10.3390/jcm14248734/s1, Table S1: PRISMA checklist; Table S2: Detailed search strategies; Table S3: List of excluded studies; Table S4: GRADE assessment; Table S5: Summary of secondary outcomes; Figure S1: HOMA-B dose comparison; Figure S2: HOMA-IR dose comparison; Figure S3: Proinsulin/insulin dose comparison.

## Abbreviations

The following abbreviations are used in this manuscript:

T2DM	Type 2 Diabetes Mellitus
GLP-1 RA	Glucagon-Like Peptide-1 Receptor Agonist
HOMA-B	Homeostatic Model Assessment of β-cell Function
HOMA-IR	Homeostatic Model Assessment of Insulin Resistance
ISR	Insulin Secretion Rate
MMTT	Mixed Meal Tolerance Test
OGTT	Oral Glucose Tolerance Test
IVGTT	Intravenous Glucose Tolerance Test
BMI	Body Mass Index
RCT	Randomized Controlled Trial
MD	Mean Difference
ROM	Ratio of Means
CI	Confidence Interval
SD	Standard Deviation
PRISMA	Preferred Reporting Items for Systematic Reviews and Meta-Analyses
PROSPERO	International Prospective Register of Systematic Reviews
DPP-4	Dipeptidyl Peptidase-4
SGLT2	Sodium-Glucose Cotransporter 2
HbA1c	Glycated Hemoglobin
SE	Standard Error
ROS	Reactive Oxygen Species
ETD	Estimated Treatment Difference
OG	Oral Glucose

## References

[1] Galicia-Garcia U., Benito-Vicente A., Jebari S., Larrea-Sebal A., Siddiqi H., Uribe K.B., Ostolaza H., Martín C. (2020). Pathophysiology of type 2 diabetes mellitus. Int. J. Mol. Sci..

[2] Hossain M.J., Al-Mamun M., Islam M.R. (2024). Diabetes mellitus, the fastest growing global public health concern: Early detection should be focused. Health Sci. Rep..

[3] Saisho Y. (2015). β-cell dysfunction: Its critical role in prevention and management of type 2 diabetes. World J. Diabetes.

[4] Bhatti J.S., Sehrawat A., Mishra J., Sidhu I.S., Navik U., Khullar N., Kumar S., Bhatti G.K., Reddy P.H. (2022). Oxidative stress in the pathophysiology of type 2 diabetes and related complications: Current therapeutics strategies and future perspectives. Free Radic. Biol. Med..

[5] De León D.D., Crutchlow M.F., Ham J.Y., Stoffers D.A. (2006). Role of glucagon-like peptide-1 in the pathogenesis and treatment of diabetes mellitus. Int. J. Biochem. Cell Biol..

[6] Lim G.E., Brubaker P.L. (2006). Glucagon-like peptide 1 secretion by the L-cell: The view from within. Diabetes.

[7] Salvador R., Moutinho C.G., Sousa C., Vinha A.F., Carvalho M., Matos C. (2025). Semaglutide as a GLP-1 agonist: A breakthrough in obesity treatment. Pharmaceuticals.

[8] Movahednasab M., Dianat-Moghadam H., Khodadad S., Nedaeinia R., Safabakhsh S., Ferns G., Salehi R. (2025). GLP-1-based therapies for type 2 diabetes: From single, dual and triple agonists to endogenous GLP-1 production and L-cell differentiation. Diabetol. Metab. Syndr..

[9] Hu S., Su X., Fan G. (2023). Efficacy and tolerability of the Subcutaneous Semaglutide for type 2 Diabetes patients: An updated systematic review and meta-analysis. Diabetol. Metab. Syndr..

[10] Luo Y., Li J.E., Zeng H., Zhang Y., Yang S., Liu J. (2025). Semaglutide alleviates the pancreatic β cell function via the METTL14 signaling and modulating gut microbiota in type 2 diabetes mellitus mice. Life Sci..

[11] Aroda V.R., Ahmann A., Cariou B., Chow F., Davies M.J., Jódar E., Mehta R., Woo V., Lingvay I. (2019). Comparative efficacy, safety, and cardiovascular outcomes with once-weekly subcutaneous semaglutide in the treatment of type 2 diabetes: Insights from the SUSTAIN 1–7 trials. Diabetes Metab..

[12] Aroda V.R., Erhan U., Jelnes P., Meier J.J., Abildlund M.T., Pratley R., Vilsbøll T., Husain M. (2023). Safety and tolerability of semaglutide across the SUSTAIN and PIONEER phase IIIa clinical trial programmes. Diabetes Obes. Metab..

[13] Rodbard H.W., Dougherty T., Taddei-Allen P. (2020). Efficacy of oral semaglutide: Overview of the PIONEER clinical trial program and implications for managed care. Am. J. Manag. Care.

[14] Husain M., Birkenfeld A.L., Donsmark M., Dungan K., Eliaschewitz F.G., Franco D.R., Jeppesen O.K., Lingvay I., Mosenzon O., Pedersen S.D. (2019). Oral semaglutide and cardiovascular outcomes in patients with type 2 diabetes. N. Engl. J. Med..

[15] Marso S.P., Bain S.C., Consoli A., Eliaschewitz F.G., Jódar E., Leiter L.A., Lingvay I., Rosenstock J., Seufert J., Warren M.L. (2016). Semaglutide and cardiovascular outcomes in patients with type 2 diabetes. N. Engl. J. Med..

[16] Thomsen R.W., Mailhac A., Løhde J.B., Pottegård A. (2025). Real-world evidence on the utilization, clinical and comparative effectiveness, and adverse effects of newer GLP-1RA-based weight-loss therapies. Diabetes Obes. Metab..

[17] Yale J.F., Bodholdt U., Catarig A.M., Catrina S., Clark A., Ekberg N.R., Erhan U., Holmes P., Knudsen S.T., Liutkus J. (2022). Real-world use of once-weekly semaglutide in patients with type 2 diabetes: Pooled analysis of data from four SURE studies by baseline characteristic subgroups. BMJ Open Diabetes Res. Care.

[18] Marassi M., Fadini G.P. (2025). Real-world evidence on oral semaglutide for the management of type 2 diabetes. A narrative review for clinical practice. Clin. Ther..

[19] Tzoulis P., Batavanis M., Baldeweg S. (2024). A real-world study of the effectiveness and safety of semaglutide for weight loss. Cureus.

[20] Perreault L., Davies M., Frias J.P., Laursen P.N., Lingvay I., Machineni S., Varbo A., Wilding J.P., Wallenstein S.O., Le Roux C.W. (2022). Changes in glucose metabolism and glycemic status with once-weekly subcutaneous semaglutide 2.4 mg among participants with prediabetes in the STEP program. Diabetes Care.

[21] Page M.J., McKenzie J.E., Bossuyt P.M., Boutron I., Hoffmann T.C., Mulrow C.D., Shamseer L., Tetzlaff J.M., Akl E.A., Brennan S.E. (2021). The PRISMA 2020 statement: An updated guideline for reporting systematic reviews. BMJ.

[22] Sterne J.A., Savović J., Page M.J., Elbers R.G., Blencowe N.S., Boutron I., Cates C.J., Cheng H.Y., Corbett M.S., Eldridge S.M. (2019). RoB 2: A revised tool for assessing risk of bias in randomised trials. BMJ.

[23] Hultcrantz M., Rind D., Akl E.A., Treweek S., Mustafa R.A., Iorio A., Alper B.S., Meerpohl J.J., Murad M.H., Ansari M.T. (2017). The GRADE Working Group clarifies the construct of certainty of evidence. J. Clin. Epidemiol..

[24] DerSimonian R., Laird N. (2015). Meta-analysis in clinical trials revisited. Contemp. Clin. Trials.

[25] Ahrén B., Masmiquel L., Kumar H., Sargin M., Karsbøl J.D., Jacobsen S.H., Chow F. (2017). Efficacy and safety of once-weekly semaglutide versus once-daily sitagliptin as an add-on to metformin, thiazolidinediones, or both, in patients with type 2 diabetes (SUSTAIN 2): A 56-week, double-blind, phase 3a, randomised trial. Lancet Diabetes Endocrinol..

[26] Ahmann A.J., Capehorn M., Charpentier G., Dotta F., Henkel E., Lingvay I., Holst A.G., Annett M.P., Aroda V.R. (2018). Efficacy and safety of once-weekly semaglutide versus exenatide ER in subjects with type 2 diabetes (SUSTAIN 3): A 56-week, open-label, randomized clinical trial. Diabetes Care.

[27] Dahl K., Brooks A., Almazedi F., Hoff S.T., Boschini C., Bækdal T.A. (2021). Oral semaglutide improves postprandial glucose and lipid metabolism, and delays gastric emptying, in subjects with type 2 diabetes. Diabetes Obes. Metab..

[28] Dwibedi C., Ekström O., Brandt J., Adiels M., Franzén S., Abrahamsson B., Rosengren A.H. (2024). Randomized open-label trial of semaglutide and dapagliflozin in patients with type 2 diabetes of different pathophysiology. Nat. Metab..

[29] Davies M., Pieber T.R., Hartoft-Nielsen M.L., Hansen O.K., Jabbour S., Rosenstock J. (2017). Effect of oral semaglutide compared with placebo and subcutaneous semaglutide on glycemic control in patients with type 2 diabetes: A randomized clinical trial. JAMA.

[30] Fan P., Ma X. (2025). Effect of Semaglutide on C-peptide levels in patients with type 2 diabetes. Pak. J. Pharm. Sci..

[31] Frías J.P., Davies M.J., Rosenstock J., Pérez Manghi F.C., Fernández Landó L., Bergman B.K., Liu B., Cui X., Brown K. (2021). Tirzepatide versus semaglutide once weekly in patients with type 2 diabetes. N. Engl. J. Med..

[32] Furusawa S., Nomoto H., Yokoyama H., Suzuki Y., Tsuzuki A., Takahashi K., Miya A., Kameda H., Cho K.Y., Takeuchi J. (2024). Glycaemic control efficacy of switching from dipeptidyl peptidase-4 inhibitors to oral semaglutide in subjects with type 2 diabetes: A multicentre, prospective, randomized, open-label, parallel-group comparison study (SWITCH-SEMA 2 study). Diabetes Obes. Metab..

[33] Sorli C., Harashima S.I., Tsoukas G.M., Unger J., Karsbøl J.D., Hansen T., Bain S.C. (2017). Efficacy and safety of once-weekly semaglutide monotherapy versus placebo in patients with type 2 diabetes (SUSTAIN 1): A double-blind, randomised, placebo-controlled, parallel-group, multinational, multicentre phase 3a trial. Lancet Diabetes Endocrinol..

[34] Rodbard H.W., Rosenstock J., Canani L.H., Deerochanawong C., Gumprecht J., Lindberg S.Ø., Lingvay I., Søndergaard A.L., Treppendahl M.B., Montanya E. (2019). Oral semaglutide versus empagliflozin in patients with type 2 diabetes uncontrolled on metformin: The PIONEER 2 trial. Diabetes Care.

[35] Korsatko S., Jensen L., Brunner M., Sach-Friedl S., Tarp M.D., Holst A.G., Heller S.R., Pieber T.R. (2018). Effect of once-weekly semaglutide on the counterregulatory response to hypoglycaemia in people with type 2 diabetes: A randomized, placebo-controlled, double-blind, crossover trial. Diabetes Obes. Metab..

[36] Kapitza C., Dahl K., Jacobsen J.B., Axelsen M.B., Flint A. (2017). Effects of semaglutide on beta cell function and glycaemic control in participants with type 2 diabetes: A randomised, double-blind, placebo-controlled trial. Diabetologia.

[37] Ji L., Dong X., Li Y., Li Y., Lim S., Liu M., Ning Z., Rasmussen S., Skjøth T.V., Yuan G. (2021). Efficacy and safety of once-weekly semaglutide versus once-daily sitagliptin as add-on to metformin in patients with type 2 diabetes in SUSTAIN China: A 30-week, double-blind, phase 3a, randomized trial. Diabetes Obes. Metab..

[38] Heise T., Mari A., DeVries J.H., Urva S., Li J., Pratt E.J., Coskun T., Thomas M.K., Mather K.J., Haupt A. (2022). Effects of subcutaneous tirzepatide versus placebo or semaglutide on pancreatic islet function and insulin sensitivity in adults with type 2 diabetes: A multicentre, randomised, double-blind, parallel-arm, phase 1 clinical trial. Lancet Diabetes Endocrinol..

[39] Mather K.J., Mari A., Heise T., DeVries J.H., Hua M., Urva S., Coskun T., Haupt A., Heine R.J., Pratt E. (2024). Effects of tirzepatide vs semaglutide on β-cell function, insulin sensitivity, and glucose control during a meal test. J. Clin. Endocrinol. Metab..

[40] Kaku K., Yamada Y., Watada H., Abiko A., Nishida T., Zacho J., Kiyosue A. (2018). Safety and efficacy of once-weekly semaglutide vs additional oral antidiabetic drugs in Japanese people with inadequately controlled type 2 diabetes: A randomized trial. Diabetes Obes. Metab..

[41] van Raalte D.H., Verchere C.B. (2016). Glucagon-like peptide-1 receptor agonists: Beta-cell protection or exhaustion?. Trends Endocrinol. Metab..

[42] Karagiannis T., Malandris K., Avgerinos I., Stamati A., Kakotrichi P., Liakos A., Vasilakou D., Kakaletsis N., Tsapas A., Bekiari E. (2024). Subcutaneously administered tirzepatide vs semaglutide for adults with type 2 diabetes: A systematic review and network meta-analysis of randomised controlled trials. Diabetologia.

[43] Rodriguez P.J., Cartwright B.M., Gratzl S., Brar R., Baker C., Gluckman T.J., Stucky N.L. (2024). Semaglutide vs tirzepatide for weight loss in adults with overweight or obesity. JAMA Intern. Med..

[44] Fonseca-Correa J.I., Correa-Rotter R. (2021). Sodium-glucose cotransporter 2 inhibitors mechanisms of action: A review. Front. Med..

