# Supplementary material for: The Effect of Semaglutide on Pancreatic β-Cell Function in Adults with Type 2 Diabetes: A Systematic Review and Meta-Analysis
Source: J Clin Med. 2025 Dec 10;14(24):8734. doi: 10.3390/jcm14248734 (PMC12733705; doi:10.3390/jcm14248734)
Supplement: Supplementary file 1 [file jcm-14-08734-s001.zip › Figures (S1-S3).pdf]

## Supplementary Figures (S1-S3)

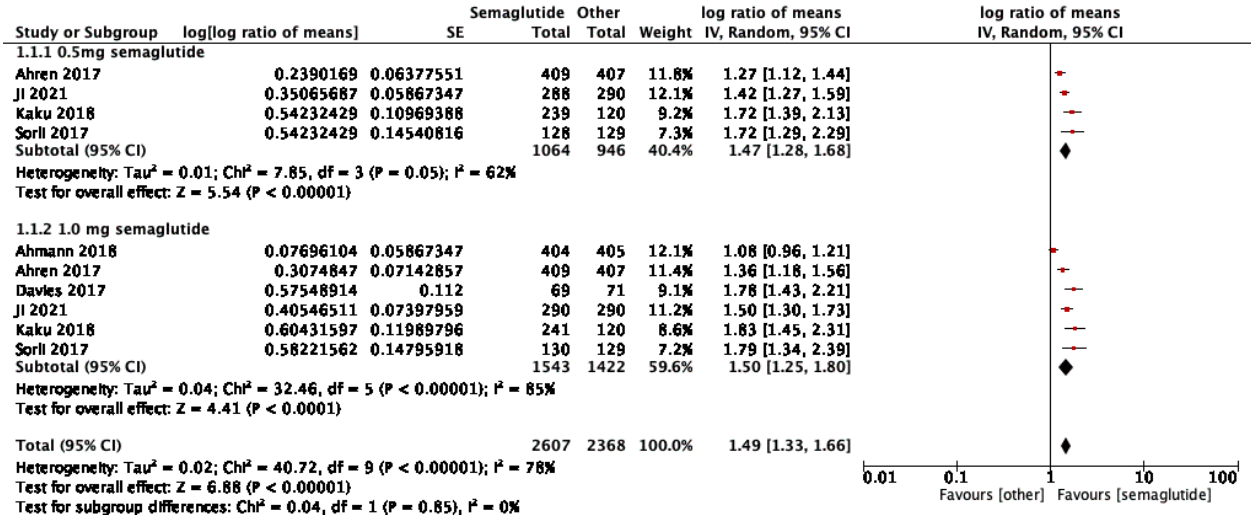


**Figure S1:** Subgroup analysis of HOMA-B by semaglutide dose (0.5 mg vs 1.0 mg)

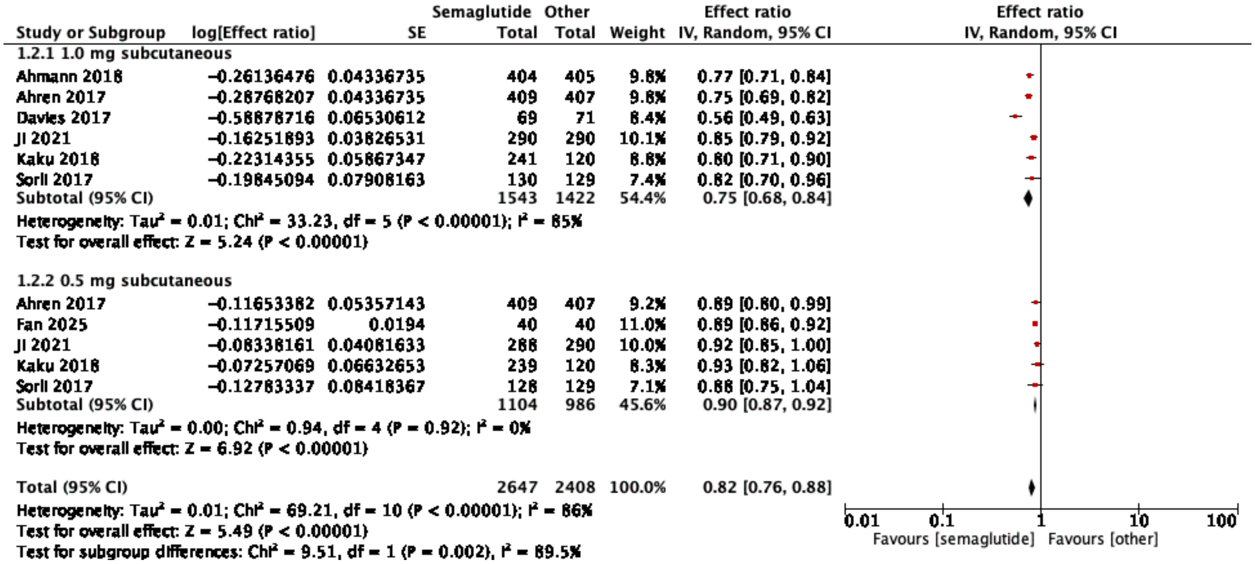


**Figure S2:** Subgroup analysis of HOMA-IR by semaglutide dose (0.5 mg vs 1.0 mg)

| Study or Subgroup | log[Treatment ratio] | SE | semaglutide Total | other Total | Weight | Treatment ratio IV, Random, 95% CI |
|---|---|---|---|---|---|---|
| **1.4.1 1.0 mg semaglutide** | | | | | | |
| Ahmann 2018 | −0.21072103 | 0.03571429 | 404 | 405 | 11.1% | 0.81 [0.76, 0.87] |
| Ahren 2017 | −0.28768207 | 0.03061224 | 409 | 407 | 11.4% | 0.75 [0.71, 0.80] |
| Ji 2021 | −0.30110509 | 0.03061224 | 290 | 290 | 11.4% | 0.74 [0.70, 0.79] |
| Kaku 2018 | −0.52763274 | 0.03061224 | 241 | 120 | 11.4% | 0.59 [0.56, 0.63] |
| Sorli 2017 | −0.40047757 | 0.04846939 | 130 | 129 | 10.4% | 0.67 [0.61, 0.74] |
| Subtotal (95% CI) | | | 1474 | 1351 | 55.8% | 0.71 [0.63, 0.79] |
| Heterogeneity: $Tau^2 = 0.02; Chi^2 = 56.60, df = 4 (P < 0.00001); I^2 = 93\%$ | | | | | | |
| Test for overall effect: $Z = 6.01 (P < 0.00001)$ | | | | | | |
| **1.4.2 0.5 mg semaglutide** | | | | | | |
| Ahren 2017 | −0.18632958 | 0.03571429 | 409 | 407 | 11.1% | 0.83 [0.77, 0.89] |
| Ji 2021 | −0.22314355 | 0.03316326 | 288 | 290 | 11.3% | 0.80 [0.75, 0.85] |
| Kaku 2018 | −0.4462871 | 0.03061224 | 239 | 120 | 11.4% | 0.64 [0.60, 0.68] |
| Sorli 2017 | −0.43078292 | 0.04846939 | 128 | 129 | 10.4% | 0.65 [0.59, 0.71] |
| Subtotal (95% CI) | | | 1064 | 946 | 44.2% | 0.73 [0.63, 0.83] |
| Heterogeneity: $Tau^2 = 0.02; Chi^2 = 44.74, df = 3 (P < 0.00001); I^2 = 93\%$ | | | | | | |
| Test for overall effect: $Z = 4.60 (P < 0.00001)$ | | | | | | |
| **Total (95% CI)** | | | 2538 | 2297 | 100.0% | 0.72 [0.66, 0.78] |
| Heterogeneity: $Tau^2 = 0.01; Chi^2 = 102.96, df = 8 (P < 0.00001); I^2 = 92\%$ | | | | | | |
| Test for overall effect: $Z = 8.04 (P < 0.00001)$ | | | | | | |
| Test for subgroup differences: $Chi^2 = 0.07, df = 1 (P = 0.78), I^2 = 0\%$ | | | | | | |

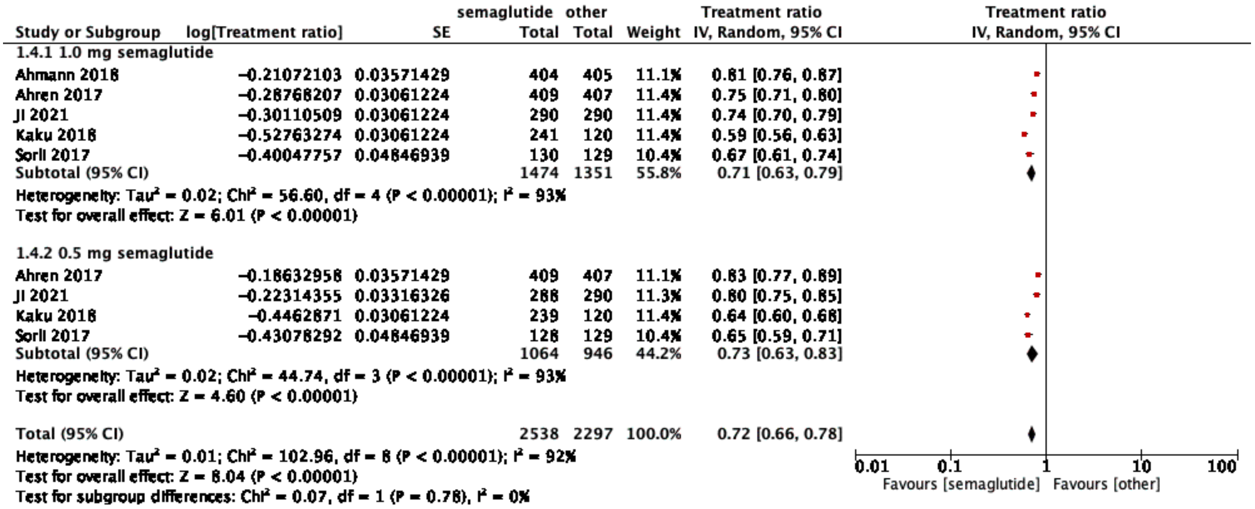


**Figure S3:** Subgroup analysis of proinsulin/insulin ratio by semaglutide dose (0.5 mg vs 1.0 mg)
