# Supplementary material for: The Effect of Semaglutide on Pancreatic β-Cell Function in Adults with Type 2 Diabetes: A Systematic Review and Meta-Analysis
Source: J Clin Med. 2025 Dec 10;14(24):8734. doi: 10.3390/jcm14248734 (PMC12733705; doi:10.3390/jcm14248734)
Supplement: Supplementary file 1 [file jcm-14-08734-s001.zip › Table S2.pdf]

Table S2. Detailed Search Strategies

Database: PubMed

Search date: April 15, 2025

Search strategy:

#1 "Semaglutide"[Mesh] OR semaglutide[tiab] OR "oral semaglutide"[tiab] OR "injectable semaglutide"[tiab]

#2 "Diabetes Mellitus, Type 2"[Mesh] OR "type 2 diabetes"[tiab] OR t2d[tiab] OR "non-insulin-dependent diabetes"[tiab]

#3 "beta-cell"[tiab] OR "β-cell"[tiab] OR "beta cell function"[tiab] OR "β-cell function"[tiab]

#4 HOMA-B[tiab] OR "HOMA B"[tiab] OR "homeostatic model assessment beta"[tiab]

#5 HOMA-IR[tiab] OR "HOMA IR"[tiab] OR "homeostatic model assessment insulin resistance"[tiab]

#6 "proinsulin insulin ratio"[tiab] OR "proinsulin-to-insulin ratio"[tiab] OR proinsulin[tiab]

#7 "insulin secretion rate"[tiab] OR ISR[tiab] OR "insulinogenic index"[tiab] OR "C-peptide"[tiab] OR "fasting insulin"[tiab] OR "disposition index"[tiab]

#8 #3 OR #4 OR #5 OR #6 OR #7

#9 #1 AND #2 AND #8

Database: Embase

Search date: April 15, 2025

Search strategy:

#1 'semaglutide'/exp OR semaglutide:ti,ab OR 'oral semaglutide':ti,ab

#2 'type 2 diabetes mellitus'/exp OR 'type 2 diabetes':ti,ab OR t2d:ti,ab

#3 'beta cell':ti,ab OR 'β-cell':ti,ab OR 'beta cell function':ti,ab OR 'β-cell function':ti,ab

#4 homa-b:ti,ab OR 'homa b':ti,ab OR 'homeostatic model assessment beta':ti,ab

#5 homa-ir:ti,ab OR 'homa ir':ti,ab OR 'homeostatic model assessment insulin resistance':ti,ab

#6 'proinsulin insulin ratio':ti,ab OR 'proinsulin-to-insulin ratio':ti,ab OR proinsulin:ti,ab

#7 'insulin secretion rate':ti,ab OR isr:ti,ab OR 'insulinogenic index':ti,ab OR 'c peptide':ti,ab OR 'fasting insulin':ti,ab OR 'disposition index':ti,ab

#8 #3 OR #4 OR #5 OR #6 OR #7

#9 #1 AND #2 AND #8

Database: Scopus

Search date: April 15, 2025

Search strategy:

```
( TITLE-ABS-KEY(semaglutide OR "oral semaglutide")
  AND
  TITLE-ABS-KEY("type 2 diabetes" OR t2d)
  AND
  TITLE-ABS-KEY(
      "beta cell" OR "β-cell" OR "beta-cell function" OR "β-cell function"
      OR HOMA-B OR "HOMA B"
      OR HOMA-IR OR "HOMA IR"
      OR "proinsulin insulin ratio" OR proinsulin
      OR "insulin secretion rate" OR ISR
      OR "insulinogenic index"
      OR "c-peptide" OR "fasting insulin"
      OR "disposition index"
    )
)
```
