# Supplementary material for: The Effect of Semaglutide on Pancreatic β-Cell Function in Adults with Type 2 Diabetes: A Systematic Review and Meta-Analysis
Source: J Clin Med. 2025 Dec 10;14(24):8734. doi: 10.3390/jcm14248734 (PMC12733705; doi:10.3390/jcm14248734)
Supplement: Supplementary file 1 [file jcm-14-08734-s001.zip › Table S3.pdf]

Table S3: List of excluded studies

Study ID	Title	Journal	Reason for Exclusion
Gentilella 2019	Glucagon-like peptide-1 receptor agonists in type 2 diabetes treatment: are they all the same?	Diabetes Metab Res Rev	Non-randomized study; narrative review, not experimental or comparative trial.
Hjerpsted 2018	Semaglutide improves postprandial glucose and lipid metabolism, and delays first-hour gastric emptying in subjects with obesity.	Diabetes Obes Metab	Wrong population: studied non-diabetic subjects with obesity.
Anyiam 2024	Metabolic effects of very-low calorie diet, Semaglutide, or combination of the two, in individuals with type 2 diabetes mellitus.	Clin Nutr	Comparator was non-pharmacological (very-low calorie diet alone).
Nomoto 2025	Improvement of β-Cell Function After Switching From DPP-4 Inhibitors to Oral Semaglutide: SWITCH-SEMA2 Post Hoc Analysis.	J Clin Endocrinol Metab	Post-hoc analysis; not a primary randomized trial.
Ji 2024	Impact of baseline characteristics on the efficacy of once-weekly subcutaneous semaglutide among participants with type 2 diabetes: A post hoc analysis of SUSTAIN China.	Diabetes Obes Metab	Post-hoc, not a primary parallel-arm randomized controlled trial.
Saisho 2020	An emerging new concept for the management of type 2 diabetes with a paradigm	Expert Opin Pharmacother	Editorial/opinion paper; lacks a randomized comparative design.

Study ID	Title	Journal	Reason for Exclusion
	shift from the glucose-centric to beta cell-centric concept of diabetes - an Asian perspective.		
Ingersen 2023	Effects of Aerobic Training and Semaglutide Treatment on Pancreatic β-Cell Secretory Function in Patients With Type 2 Diabetes.	J Clin Endocrinol Metab	Comparator is exercise alone, not a standard anti-diabetic pharmacological agent.
Doggrell 2018	Semaglutide in type 2 diabetes - is it the best glucagon-like peptide 1 receptor agonist (GLP-1R agonist)?	Expert Opin Drug Metab Toxicol	Non-experimental review/opinion article; not primary research.
Gojani 2025	Single and Combined Impact of Semaglutide, Tirzepatide, and Metformin on β-Cell Maintenance and Function Under High-Glucose-High-Lipid Conditions: A Comparative Study.	Int J Mol Sci	Non-clinical population: used in vitro or non-standard study populations.
Bruinstroop 2018	Retrospective Analysis of an Insulin-to-Liraglutide Switch in Patients with Type 2 Diabetes Mellitus.	Diabetes Ther	Wrong intervention: did not involve semaglutide as an intervention group.
Doggrell 2020	Will oral semaglutide be used to reduce cardiovascular risk in subjects with type 2 diabetes instead of subcutaneous semaglutide?	Expert Opin Biol Ther	Comparator is another semaglutide arm only; not suitable per comparator exclusion.

Study ID	Title	Journal	Reason for Exclusion
Shao 2024	Effects of GLP-1 Receptor Agonist on Glycolipid Metabolism and Micro Inflammatory Status in Patients with Abdominal Obesity and Type 2 Diabetes.	Altern Ther Health Med	Intervention does not include semaglutide as required.
BuschCBE 2024	Recellularization via electroporation therapy of the duodenum combined with glucagon-like peptide-1 receptor agonist to replace insulin therapy in patients with type 2 diabetes: 12-month results of a first-in-human study.	Gastrointest Endosc	Includes non-standard experimental therapy (electroporation); confounds semaglutide.
Aoyama 2024	Effects of weight loss from oral semaglutide administration on cardiometabolic risk factors in Japanese patients with type 2 diabetes: a retrospective analysis using propensity score matching.	Diabetol Int	Outcomes assessed do not include β-cell function or direct glycemic endpoints.
Ren 2025	The effect of semaglutide combined with metformin on liver inflammation and pancreatic beta-cell function in patients with type 2 diabetes and non-alcoholic fatty liver disease.	J Diabetes Complications	Non-randomized or unclear study design; post-hoc or observational.
Jones 2016	Markers of β-Cell Failure Predict Poor Glycemic Response to GLP-1	Diabetes Care	Interventions did not use semaglutide for

Study ID	Title	Journal	Reason for Exclusion
	Receptor Agonist Therapy in Type 2 Diabetes.		β-cell function assessment.
Aroda 2020	Impact of baseline characteristics and beta-cell function on the efficacy and safety of subcutaneous once-weekly semaglutide: A patient-level, pooled analysis of the SUSTAIN 1-5 trials.	Diabetes Obes Metab	Post-hoc pooled analysis; does not meet primary RCT criteria.
Miyasaka 2022	[New drug for type 2 diabetes: introduction of oral Semaglutide (Rybelsus®) tablets, an oral GLP-1 receptor agonist].	Nihon Yakurigaku Zasshi	Language exclusion: full text not available in English.
Gregorić 2025	Semaglutide improved sperm morphology in obese men with type 2 diabetes mellitus and functional hypogonadism	Diabetes, Obesity and Metabolism	Population includes non-standard selection (obese men with hypogonadism).
Sashi 2025	Clinical Analysis of Resemblance and Dissimilarities of Glucagon-like Peptide-1 Receptor Agonists: Therapeutic Approach Towards the Management of Diabetes Mellitus.	Current Drug Therapy	Non-randomized; narrative analysis, not a clinical comparative trial.
Sohn 2024	Long-term comparison of renal and metabolic outcomes after sodium–glucose co-transporter 2 inhibitor or glucagon-like peptide-1 receptor	BMC Medicine	Interventions did not include semaglutide or comparator was not appropriate.

Study ID	Title	Journal	Reason for Exclusion
	agonist therapy in type 2 diabetes		
Al-Omar 2024	Budget Impact Analysis for Semaglutide, Tirzepatide, and Dulaglutide for Type 2 Diabetes Mellitus Management in Saudi Arabia	Value in Health	Outcomes assessed are health economics, not efficacy or β-cell function.
Kalinkova 2024	Pharmacogenetic markers for personalized treatment in patients with type 2 diabetes mellitus and obesity	Obesity Facts	Did not assess semaglutide intervention per PICO; focused on genetic predictors.
Maldonado 2024	Tirzepatide reduces HbA1c and body weight significantly more than placebo or semaglutide irrespective of baseline beta-cell function: post-hoc analysis from SURPASS-1 and SURPASS-2	Diabetologie und Stoffwechsel	Post-hoc analysis with non-semaglutide-focused interventions for β-cell outcomes.
Rodríguez Jiménez 2024	Transforming body composition with semaglutide in adults with obesity and type 2 diabetes mellitus	Frontiers in Endocrinology	Outcomes are limited to body composition; lacks β-cell function focus.
Li 2024	Efficacy and safety of semaglutide combined with metformin in treating T2DM with overweight or obesity: a systematic review and meta-analysis	American Journal of Translational Research	Non-randomized meta-analysis; lacks direct comparative trial data.
Lin 2023	Empagliflozin monotherapy in non-	medRxiv	Intervention does not include semaglutide.

Study ID	Title	Journal	Reason for Exclusion
	alcoholic fatty liver disease with type 2 diabetes: study protocol for a randomised controlled clinical trial		
Arslanian 2023	Improvement in insulin sensitivity and glucose metabolism in adolescents with obesity treated with once-weekly semaglutide 2.4 mg: a secondary analysis of the STEP TEENS trial	Hormone Research in Paediatrics	Excluded population: pediatric/adolescent participants only.
Xu 2023	Effect of glucagon-like peptide-1 receptor agonist on insulin secretion index and serum Wnt5a protein in patients with new-onset type 2 diabetes mellitus	Journal of Diabetes and Metabolic Disorders	Non-semaglutide GLP-1RA intervention used.
Anyiam 2023	The Effect of Combining Very-Low-Calorie Diet with Semaglutide on Beta-Cell Function in Individuals with Type 2 Diabetes	Diabetes	Non-pharmacological comparator (diet only) or outcome not β-cell function.
Crabtree 2023	The impact of diabetes duration on HbA1c and weight changes associated with injectable semaglutide: Subanalysis from the Association of British Clinical Diabetologist (ABCD) Semaglutide Audit	Diabetic Medicine	Outcome does not include β-cell function assessment.
Berra 2023	Real world effectiveness of subcutaneous	Frontiers in Endocrinology	Retrospective observational design;

Study ID	Title	Journal	Reason for Exclusion
	semaglutide in type 2 diabetes: A retrospective, cohort study (Sema-MiDiab01)		not a randomized trial.
Kim 2023	The Efficacy of Treatment Intensification by Quadruple Oral Therapy Compared to GLP-1RA Therapy in Poorly Controlled Type 2 Diabetes Mellitus Patients: A Real-World Data Study	Diabetes and Metabolism Journal	Intervention group is not semaglutide; comparator inappropriate as per PICO.
Kalinkova 2023	Pharmacogenetics of Glucagon-like-peptide-1 receptor in diabetes management	Pharmacia	Non-randomized; focused on genetic predictors, not semaglutide outcomes.
Volpe 2023	Oral semaglutide improves body composition and preserves lean mass in patients with type 2 diabetes: a 26-week prospective real-life study	Frontiers in Endocrinology	Non-randomized, prospective (observational) design.
Mather 2022	Tirzepatide improves multiple aspects of beta cell function	Diabetologia	Interventions did not use semaglutide; not relevant to PICO.
Mari 2022	Tirzepatide Improves Multiple Aspects of Beta-Cell Function	Diabetes	Intervention is not semaglutide.
Pi 2022	Clinical Efficacy Evaluation and Long-Term Prognosis of Glucagon-Like Peptide-1 Combined with Sodium	Indian Journal of Pharmaceutical Sciences	Semaglutide population not clearly described; mixed interventions.

Study ID	Title	Journal	Reason for Exclusion
	Glucose Cotransporter-2 Inhibitor in Diabetes		
Montanya 2021	Glycaemic variability of oral semaglutide vs empagliflozin: A post-hoc analysis of PIONEER 2	Diabetologia	Post-hoc analysis, not primary parallel RCT.
Gavigan 2021	Addition of a GLP-1 agonist to insulin therapy in type 2 diabetes	Diabetes	GLP-1RA intervention not specified as semaglutide.
Balcazar 2021	Semaglutide Once Weekly In Persons with Type 2 Diabetes: Real-World Analysis of the Colombian Diabetes Registry (COL-REAL 1 Study)	Metabolism: Clinical and Experimental	Outcomes not relevant to β-cell function; design unclear or non-randomized.
Giorgino 2019	Effect of once-weekly dulaglutide versus insulin glargine in people with type 2 diabetes and different baseline glycaemic patterns: A post hoc analysis of the AWARD-2 clinical trial	Diabetes, Obesity and Metabolism	Comparator inappropriate; does not involve semaglutide intervention.
Fischli 2017	Update on therapy with GLP-1-receptor agonists in patients with type 2 diabetes	Therapeutische Umschau	Non-randomized design; narrative review.
Fonseca 2017	Semaglutide-induced reductions in insulin resistance are mediated primarily via weight loss in subjects with type 2 diabetes (SUSTAIN 1-3)	Diabetologia	Post-hoc analysis, not a standalone randomized comparative trial.

Study ID	Title	Journal	Reason for Exclusion
Wu 2017	Exendin-4 promotes pancreatic β-cell proliferation via inhibiting the expression of Wnt5a	Endocrine	Intervention is not semaglutide; preclinical population.
Jiang 2024	Clinical observation of semaglutide combined with rosiglitazone in treatment of type 2 diabetes	Drugs and Clinic	Language exclusion: full text not available in English.
Movahednasab 2025	GLP-1-based therapies for type 2 diabetes: from single, dual and triple agonists to endogenous GLP-1 production and L-cell differentiation	Diabetology and Metabolic Syndrome	Narrative review or non-randomized study.
Jang 2024	Fasting GLP-1 Levels and Albuminuria Are Negatively Associated in Patients with Type 2 Diabetes Mellitus	Journal of Personalized Medicine	Study setting not clinical efficacy; wrong population or outcomes.
Siewe 2024	A mathematical model of obesity-induced type 2 diabetes and efficacy of anti-diabetic weight reducing drug	Journal of Theoretical Biology	Non-clinical, model-based study; not randomized human trial.
Takebayashi 2024	Exacerbation in Glycemic Control by Switching from Subcutaneous Dulaglutide Injection to Oral Semaglutide Administration in a Patient with Type 2 Diabetes, Who Had Undergone Gastrectomy	Dokkyo Journal of Medical Sciences	Excluded population: post-surgical (gastrectomy); not generalizable to T2D.

Study ID	Title	Journal	Reason for Exclusion
Viggers 2023	Effects of Incretin Therapy on Skeletal Health in Type 2 Diabetes—A Systematic Review	JBMR Plus	Systematic review, not a primary randomized study.
Cho 2023	Glucagon-like peptide-1 therapy for youth with type 2 diabetes	Journal of Diabetes Investigation	Excluded population: pediatric patients only.
Denimal 2023	Liraglutide reduces plasma dihydroceramide levels in patients with type 2 diabetes	Cardiovascular Diabetology	Intervention is liraglutide, not semaglutide.
Arai 2022	Efficacy and safety of oral semaglutide in patients with non-alcoholic fatty liver disease complicated by type 2 diabetes mellitus: A pilot study	JGH Open	Pilot non-randomized study; lacks proper control/comparator.
Zhao 2021	GLP-1 Receptor Agonists: Beyond Their Pancreatic Effects	Frontiers in Endocrinology	Narrative review, not a primary trial.
Tanday 2021	Amplifying the antidiabetic actions of glucagon-like peptide-1: Potential benefits of new adjunct therapies	Diabetic Medicine	Non-randomized; adjunct therapies review, not semaglutide-focused RCT.
Rathmann 2017	Impact of insulin sensitivity, beta-cell function and glycaemic control on initiation of second-line glucose-lowering treatment in newly diagnosed type 2 diabetes	Diabetes, Obesity and Metabolism	Interventions do not focus on semaglutide; mixed therapies.

Study ID	Title	Journal	Reason for Exclusion
Yu 2020	The Efficacy and Safety of Basic Insulin Combined with GLP-1 Receptor Agonist in the Treatment of Type 2 Diabetes Mellitus	Chinese Journal of Pharmaceutical Biotechnology	Mixed interventions; GLP-1RA not specified as semaglutide.
Ikushima 2018	A Randomized Trial Investigating the Pharmacokinetics, Pharmacodynamics, and Safety of Subcutaneous Semaglutide Once-Weekly in Healthy Male Japanese and Caucasian Subjects	Advances in Therapy	Excluded population: healthy subjects, not patients with T2D.
Røder 2019	Clinical potential of treatment with semaglutide in type 2 diabetes patients	Drugs in Context	Narrative review; not a randomized, controlled clinical trial.
Ahrén 2019	Glucagon-like peptide-1 receptor agonists for type 2 diabetes: A rational drug development	Journal of Diabetes Investigation	Non-randomized; drug development review.
Lambert 2016	GLP-1 receptor agonists in the management of Type 2 Diabetes	SA Pharmaceutical Journal	Non-randomized; review of drug class, not clinical trial.
Schernthaner-Reiter 2016	Combination therapy of SGLT2 inhibitors with incretin-based therapies for the treatment of type 2 diabetes mellitus: Effects and mechanisms of action	Expert Review of Endocrinology and Metabolism	Non-randomized; mechanistic review, not clinical trial.
Ampudia-Blasco 2016	Treatment of diabetes mellitus (II). Non-insulin hypoglycemic agents	Medicine (Spain)	Review article summarizing non-insulin

Study ID	Title	Journal	Reason for Exclusion
			pharmacological agents
