# Supplementary material for: The Effect of Semaglutide on Pancreatic β-Cell Function in Adults with Type 2 Diabetes: A Systematic Review and Meta-Analysis
Source: J Clin Med. 2025 Dec 10;14(24):8734. doi: 10.3390/jcm14248734 (PMC12733705; doi:10.3390/jcm14248734)
Supplement: Supplementary file 1 [file jcm-14-08734-s001.zip › Table S4.pdf]

Table S4: GRADE assessment

Outcome	No. of Studies	Study Design	Risk of Bias	Inconsistency	Indirectness	Imprecision	Other Considerations	No. of Patients (Semaglutide)	No. of Patients (Comparator)	Effect (Relative, 95% CI)	Certainty	Importance
**HOMA-B**	6	RCT	Serious[a]	Serious[b]	Not serious	Not serious	Publication bias strongly suspected[c]	1713	1712	Log effect ratio 1.50 (1.25 to 1.80)	⊕◯◯◯ Very low[a,b,c]	**Critical**
**HOMA-IR**	8	RCT	Serious[d]	Serious[e]	Not serious	Not serious	Publication bias strongly suspected[c]	1753	1752	Log effect ratio 0.82 (0.73 to 0.94)	⊕◯◯◯ Very low[c,d,e]	**Critical**
**Proinsulin / Insulin**	5	RCT	Serious[f]	Serious[g]	Not serious	Not serious	Publication bias strongly suspected[c]	1233	1231	Log effect ratio 0.70 (0.63 to 0.79)	⊕◯◯◯ Very low[c,f,g]	**Critical**

CI: confidence interval

Explanations

a. 5 of 6 studies had high or unclear risk, including incomplete data and lack of assessor blinding

b. Downgraded one level for inconsistency: $I^2 = 85\%$ with one moderate outlier, but all studies favored the intervention, and the overall effect was statistically significant.

c. <10 studies; potential for missing negative studies

d. Downgraded one level for serious risk of bias: 5 of 8 studies contributing to this outcome had high or unclear risk across multiple domains, including blinding and incomplete outcome data.

e. Downgraded one level for serious inconsistency: substantial heterogeneity ($I^2 = 95\%$) in effect estimates across studies, not fully explained by study design or population differences.

f. 3 of 4 studies had high or unclear risk, including incomplete data and lack of assessor blinding

g. Downgraded one level for inconsistency: $I^2 = 93\%$ indicates severe heterogeneity, although effect sizes were directionally consistent

We assessed the certainty of evidence for each outcome using the GRADE approach, implemented in GRADEpro GDT. For all three key outcomes (HOMA-B, HOMA-IR, and Proinsulin-to-Insulin ratio), the certainty of evidence was rated as very low, primarily due to concerns related to risk of bias, inconsistency, and suspected publication bias.

- HOMA-B (6 RCTs): The pooled log effect ratio was 1.50 (95% CI: 1.25 to 1.80). We downgraded the certainty of evidence for serious risk of bias (due to lack of blinding and incomplete outcome data in several studies), serious inconsistency ($I^2 = 85$), and strongly suspected publication bias due to inclusion of fewer than 10 studies. Certainty of evidence: Very Low.
- HOMA-IR (8 RCTs): The pooled log effect ratio was 0.82 (95% CI: 0.73 to 0.94). We downgraded for serious risk of bias, very serious inconsistency ($I^2 = 95\%$), and strongly suspected publication bias. Certainty of evidence: Very Low.
- Proinsulin-to-Insulin ratio (5 RCTs): The pooled treatment ratio was 0.70 (95% CI: 0.63 to 0.79). Downgrades were applied for serious risk of bias, serious inconsistency ($I^2 = 93\%$), and strongly suspected publication bias. Certainty of evidence: Very Low.

All outcomes were considered critical to evaluating β-cell function or insulin resistance, and no upgrades were applied. Absolute effects were not calculated due to the continuous nature of these outcomes.
