# Supplementary material for: The Effect of Semaglutide on Pancreatic β-Cell Function in Adults with Type 2 Diabetes: A Systematic Review and Meta-Analysis
Source: J Clin Med. 2025 Dec 10;14(24):8734. doi: 10.3390/jcm14248734 (PMC12733705; doi:10.3390/jcm14248734)
Supplement: Supplementary file 1 [file jcm-14-08734-s001.zip › Table S5.pdf]

Table S5: Summary of secondary outcomes reported in included studies (ISR, insulinogenic index, β-cell glucose sensitivity, C-peptide, fasting insulin, proinsulin, and disposition index).

Study ID	Outcome Measured	Assessment Method	Intervention	Comparator	Direction of Effect	Summary / Notes
Kapitza 2017 **[36]**	Insulin Secretion Rate (ISR)	IVGTT	Semaglutide	Placebo	↑ Improved	Marked improvement in early and late ISR phases.
Heise 2022 **[38]**	Insulin Secretion Rate (ISR)	IVGTT / Clamp	Semaglutide	Tirzepatide; Placebo	↑ Improved	Semaglutide improved ISR; tirzepatide produced even greater increases.
Mather 2024 **[39]**	Insulin Secretion Rate (ISR)	MMTT	Semaglutide	Tirzepatide; Placebo	↑ Improved	ISR doubled with semaglutide; tirzepatide showed larger increases; ISR declined with placebo.
Dahl 2021 **[27]**	β-cell glucose sensitivity	Mixed Meal Tests (standard and fat-rich)	Semaglutide oral	Placebo	↑ Improved	Significant increases in β-CGS; semaglutide improved β-cell responsiveness.
Mather 2024 **[39]**	β-cell glucose sensitivity	MMTT + Clamp	Semaglutide	Tirzepatide	↑ Improved	Both drugs increased β-CGS; tirzepatide showed larger clamp-derived effects.

Dwibedi 2024 **[28]**	Insulinogenic Index	OGTT / Mixed phenotyping	Semaglutide	Dapagliflozin	↑ Improved	Greater improvements in GLP-1–naïve patients and those with BMI >30 kg/m².
Sorli 2017 **[33]**	C-peptide	Fasting	Semaglutide	Placebo	↑ Improved	Significant increases for both 0.5 mg and 1.0 mg semaglutide.
Kapitza 2017 **[36]**	C-peptide	IVGTT	Semaglutide	Placebo	↑ Improved	Fasting and stimulated C-peptide improved; postprandial unchanged.
Ahrén 2017 **[25]**	C-peptide	Fasting	Semaglutide	Sitagliptin	↑ Improved	Improvement observed mainly with 0.5 mg dose.
Ji 2021 **[37]**	C-peptide	Fasting	Semaglutide	Sitagliptin	↑ Improved	Consistent improvements across doses.

Legend: ISR—insulin secretion rate; β-CGS—β-cell glucose sensitivity; MMTT—mixed meal tolerance test; IVGTT—intravenous glucose tolerance test; OGTT—oral glucose tolerance test; AUC—area under the curve; DI—disposition index; OAD—oral antidiabetic drug; s.c.—subcutaneous.
